# Broad host range phages target global *Clostridium perfringens* bacterial strains and clear infection in five-strain model systems

**DOI:** 10.1128/spectrum.03784-23

**Published:** 2024-03-21

**Authors:** Anisha M. Thanki, Emmanuel K. Osei, Natasha Whenham, Michael G. Salter, Mike R. Bedford, Helen V. Masey O’Neill, Martha R. J. Clokie

**Affiliations:** 1 Department of Genetics and Genome Biology, Leicester Centre for Phage Research, University of Leicester, Leicester, United Kingdom; 2 Department of Agriculture, Food and the Marine, Teagasc Food Research Centre, Moorepark, Ireland; 3 APC Microbiome Ireland, University College, Cork, Ireland; 4 AB Agri, Innovation Way, Peterborough Business Park, Peterborough, United Kingdom; 5 AB Vista, Woodstock Court, Marlborough Business Park, Marlborough, Wiltshire, United Kingdom; University of Pittsburgh School of Medicine, Pittsburgh, Pennsylvania, USA

**Keywords:** bacteriophage therapy, *Clostridium perfringens*, phage cocktails, bacteriophages, *Galleria mellonella*

## Abstract

*Clostridium perfringens* is a prevalent bacterial pathogen in poultry, and due to the spread of antimicrobial resistance, alternative treatments are needed to prevent and treat infection. Bacteriophages (phages), viruses that kill bacteria, offer a viable option and can be used therapeutically to treat *C. perfringens* infections. The aim of this study was to isolate phages against *C. perfringens* strains currently circulating on farms across the world and establish their virulence and development potential using host range screening, virulence assays, and larva infection studies. We isolated 32 phages of which 19 lysed 80%–92% of our global *C. perfringens* poultry strain collection (*n* = 97). The virulence of these individual phages and 32 different phage combinations was quantified in liquid culture at multiple doses. We then developed a multi-strain *C. perfringens* larva infection model, to mimic an effective poultry model used by the industry. We tested the efficacy of 16/32 phage cocktails in the larva model. From this, we identified that our phage cocktail consisting of phages CPLM2, CPLM15, and CPLS41 was the most effective at reducing *C. perfringens* colonization in infected larvae when administered before bacterial challenge. These data suggest that phages do have significant potential to prevent and treat *C. perfringens* infection in poultry.

**IMPORTANCE:**

*Clostridium perfringens* causes foodborne illness worldwide, and 95% of human infections are linked to the consumption of contaminated meat, including chicken products. In poultry, *C. perfringens* infection causes necrotic enteritis, and associated mortality rates can be up to 50%. However, treating infections is difficult as the bacterium is becoming antibiotic-resistant. Furthermore, the poultry industry is striving toward reduced antibiotic usage. Bacteriophages (phages) offer a promising alternative, and to progress this approach, robust suitable phages and laboratory models that mimic *C. perfringens* infections in poultry are required. In our study, we isolated phages targeting *C. perfringens* and found that many lyse *C. perfringens* strains isolated from chickens worldwide. Consistent with other published studies, in the model systems we assayed here, when some phages were combined as cocktails, the infection was cleared most effectively compared to individual phage use.

## INTRODUCTION

*Clostridium perfringens* is an anaerobic enteric pathogen and one of the most common causes of human foodborne illness (1). In the USA, *C. perfringens* is responsible for~1 million infections, and in the EU, ~5 million infections annually. Furthermore,~95% of acute gastrointestinal infections are linked to the consumption of contaminated meat, including poultry products (2). Disease pathogenesis stems from the infecting *C. perfringens* strain’s ability to produce more than 20 pathogenic enzymes and toxins. Strains are classified into seven toxin types from A to G, based on toxin carriage and strain, types A, C, and G are frequently linked to human infections (3).

In poultry, *C. perfringens* is a causative agent of necrotic enteritis (NE), a common bacterial disease in modern broiler flocks caused by toxins produced by type A and G strains (4). Disease in poultry is characterized by necrotic lesions in the bird’s small intestinal mucosa (5), and clinical NE is commonly seen in 2–5-week-old birds with symptoms including depression and decreased appetite. Mortality rates range from 2% to 50% (6). Subclinical NE disease is also common and results in significant economic losses due to suboptimal growth performance and loss of feed conversion efficiency. The disease costs the international poultry industry in excess of US $6 billion per year in production losses (7).

Common antibiotics used to treat the infection in poultry include virginiamycin, bacitracin, and lincomycin. These are broad-spectrum, disrupt the birds’ normal microbiota leading to further complications, and contribute to creating a reservoir of antibiotic-resistant bacteria (8). Bacteriophages (phages), viruses that kill bacteria, offer a feasible alternative to antibiotics. Phages are bacterial species-specific and when used therapeutically specifically target the pathogen of interest and avoid the disruption of the normal flora unlike antibiotics (9–11).

Phages used for therapeutic application are first characterized according to their host range to determine which target strains they can lyse. Ideal phages for therapy are those that infect multiple target strains. For example, phage BG3P lysed 29/32 *C*. *perfringens* strains isolated in China (12) and would, therefore, be considered an ideal candidate to treat strains in this region whereas phage vB_CP_qdyz_lysed only 4/21 Chinese *C. perfringens* strains so is less useful (13). A common theme with published studies is that they screen phage host range against regionally local *C. perfringens* strains rather than strains from across the world, and consequently, the relevance of the phage on a global scale is not addressed.

Host range screening provides essential information on which strains the phages could lyse, but it does not provide data on the efficacy of killing, an activity that is assessed in virulence assays and *Galleria mellonella* larva infection studies (14, 15). Local virulence is a high-throughput quantitative method that quantifies phage virulence against target strains (14). Various doses of phage are assessed for their ability to reduce bacterial numbers, and such data allow for direct virulence comparison between phages and are referred to as a virulence index. The larval infection model uses *G. mellonella* as this moth shares a similar basic innate immune response with mammals (15). Both methods allow for high-throughput screening of phage efficacy, which subsequently informs on which phage or phage cocktails should be used and at what doses in subsequent animal studies. However, such models have not been developed to study *C. perfringens* phages to date, and hence, the objective of this work was to use these tools to inform on the selection of optimal phages for therapy.

The first aim was to collect phages against *C. perfringens* strains from infected poultry around the world and develop *C. perfringens* models to study and compare the efficacy of phage virulence. We isolated 32 phages and assessed their virulence individually and as cocktails using both a local virulence assay and a *C. perfringens* larva infection model. The larva model we developed is based on a five-strain challenge poultry model used to experimentally induce NE as our target was to develop a model similar to the one used in poultry (16).

## RESULTS

### Isolation of *C. perfringens* phages

From 159 enriched environmental samples, 32 phages were isolated on *C. perfringens* host strains D2, U5, and CP42 (Table 1). Cow fecal material and slurry/silage were the best sources for *C. perfringens* phages from which 18 phages were isolated. The remaining phages were isolated from chicken and sheep feces and waste-water samples.

**TABLE 1:** List of *C. perfringens* phages isolated

Phage name	Description of the sample phage was isolated from	*C. perfringens* host strain	Phage morphology based on TEM
CPLM1	Cow feces, farm 1, Leicestershire	D2	Myovirus
CPLM2	Cow feces, farm 1, Leicestershire	D2	Myovirus
CPLM7	Cow feces, farm 1, Leicestershire	D2	Myovirus
CPLM8	Cow slurry, farm 1, Leicestershire	D2	Myovirus
CPLM13	Cow feces, farm 1, Leicestershire	D2	Myovirus
CPLM14	Silage, farm 1, Leicestershire	D2	Myovirus
CPLM15	Cow slurry, farm 1, Leicestershire	D2	Myovirus
CPLM16	Cow feces, farm 1, Leicestershire	D2	Myovirus
CPLM17	Cow slurry, farm 1, Leicestershire	D2	Myovirus
CPLM18	Cow slurry, farm 1, Leicestershire	D2	Myovirus
CPLM21	Dirty puddle, farm 1, Leicestershire	D2	Myovirus
CPLS24	Chicken feces, farm 2, Yorkshire	U5	Siphovirus
CPLS25	Sheep feces, farm 3, Leicestershire	U5	Siphovirus
CPLS26	Chicken feces, farm 4, Yorkshire	U5	Siphovirus
CPLS27	Cow slurry, farm 1, Leicestershire	U5	Siphovirus
CPLS29	Drain sample, farm 5, Yorkshire	U5	Siphovirus
CPLS30	Silage, farm 1, Leicestershire	U5	Siphovirus
CPLS31	Sheep feces, farm 3, Leicestershire	U5	Siphovirus
CPLS32	Chicken feces, farm 6, Yorkshire	U5	Siphovirus
CPLS34	Chicken feces, farm 7, Yorkshire	U5	Siphovirus
CPLS35	Chicken feces, farm 7, Yorkshire	U5	Siphovirus
CPLS38	Chicken feces, farm 6, Yorkshire	U5	Siphovirus
CPLS39	Chicken feces, farm 6, Yorkshire	U5	Siphovirus
CPLS40	Water sample, farm 6, Yorkshire	U5	Siphovirus
CPLS41	Chicken feces, farm 7, Yorkshire	U5	Siphovirus
CPLS42	Cow feces, farm 1, Leicestershire	U5	Siphovirus
CPLS43	Dirty puddle, farm 1, Leicestershire	U5	Siphovirus
CPLS44	Cow feces, farm 1, Leicestershire	U5	Siphovirus
CPLS45	Cow feces, farm 1, Leicestershire	U5	Siphovirus
CPLP46	Cow feces, farm 8, Leicestershire	CP42	Podovirus
CPLS47	Cow feces, farm 8, Leicestershire	CP42	Siphovirus
CPLS48	Cow feces, farm 8, Leicestershire	CP42	Siphovirus

**Fig 1:**
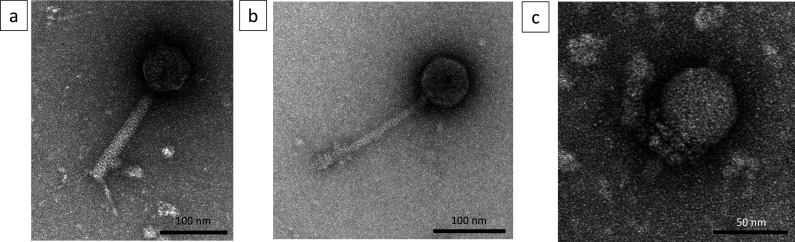
TEM images of the phages isolated within the phage collection. Image (**a**) shows the structure of the CPLM myoviruses, (**b**) shows the structure of the CPLS siphoviruses, and (**c**) shows the structure of the CPLP podovirus.

Phages were visualized on the transmission electron microscope (TEM), and based on their morphology within the collection, there are 11 myoviruses, 20 siphoviruses, and 1 podovirus (Fig. 1). Phages were named using the following scheme: phage CPLM means ***C***. *perfringens* **P**hage isolated at **L**eicester **M**yovirus structure, CPL**S** means **S**iphovirus structure, and CPL**P** means **P**odovirus structure.

### Host range of *C. perfringens* phages

**Fig 2:**
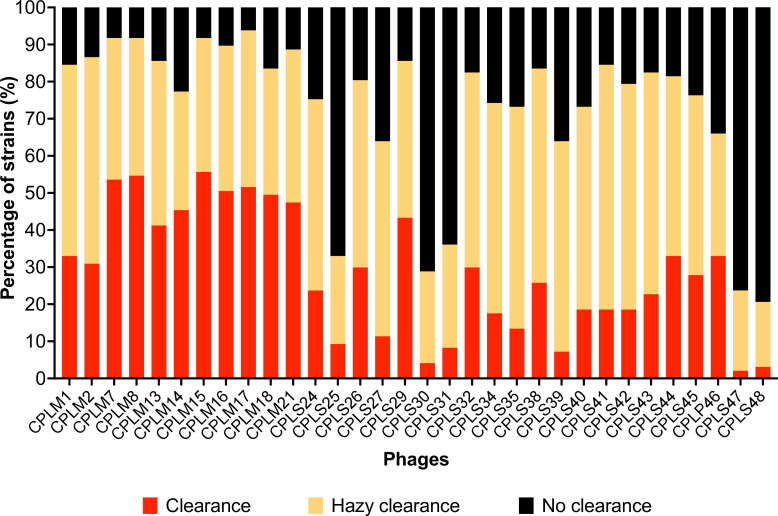
Percentage of *C. perfringens* strains (*n* = 97) lysed by individual phages.

The host range of 32 phages was tested against a panel of 97 *C*. *perfringens* strains, isolated across the world from infected chickens. Figure 2 highlights the percentage of strains the phages lysed seen as either clearance or hazy clearing on spot tests. The full host range table is shown in Table S1.

Phages CPLM7, CPLM8, CPLM15, CPLM16, and CPLM17 were the broadest and infected over 90% of strains. Other CPLM phages, CPLS24, CPLS26, CPLS29, CPLS32, CPLS38, CPLS41, CPLS42, CPLS43, and CPLS44, also had broad host ranges and infected 80% of the strains screened. In comparison, phages CPLS25, CPLS30, CPLS31, CPLS47, and CPLS48 had the narrowest host range and only infected ~30% of the strains tested. Overall, there was at least one phage within the collection that could infect each *C. perfringens* strain screened (Table S1).

### pH and temperature stability of *C. perfringens* phages

The pH and temperature stability were tested for the 19 phages that lysed over 80% of the strain collection as they were the broadest and, thus, are potential candidates for therapy. The phages tested were CPLM1, CPLM2, CPLM7, CPLM8, CPLM13, CPLM15, CPLM16, CPLM17, CPLM18, CPLM21, CPLS24, CPLS26, CPLS29, CPLS32, CPLS38, CPLS41, CPLS42, CPLS43, and CPLS44. All phages were fully stable from pH 4 to 11. CPLS phages retained activity at pH 3, but there was a 20%–40% reduction in plaquing efficiency (Table S2).

**Fig 3:**
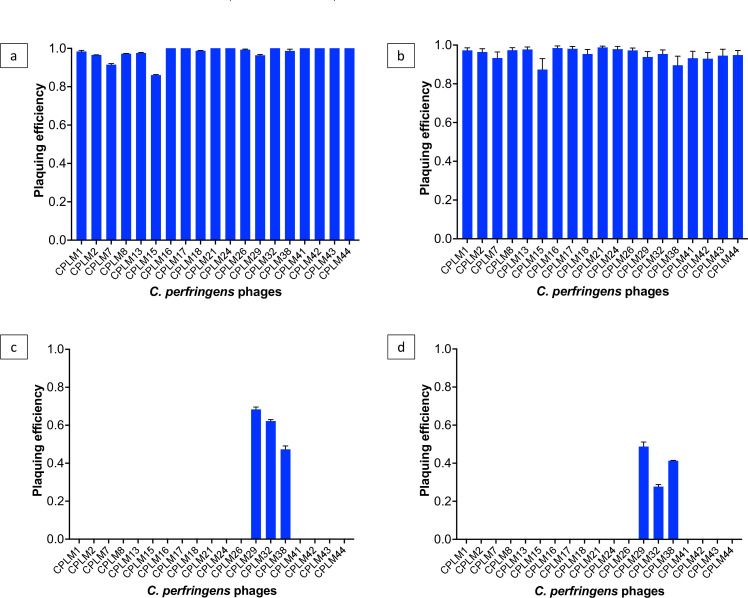
Temperature stability of *C. perfringens* phages at (**a**) 40°C and 50°C, (**b**) 60°C, (**c**) 70°C, and (**d**) 80°C. Plaquing efficiency was compared to phages stored at 4°C, and data plotted are averages from three biological replicates, each with three replicates. Error bars show the standard error of the mean.

With respect to temperature, all phages remained stable at 40°C, 50°C, and 60°C for 1 hour with no loss in phage titer (Fig. 3a and b). At 70°C, only phages CPLS29, CPLS32, and CPLS38 retained activity, but their plaquing efficiencies were reduced by 30%, 40%, and 50%, respectively (Fig. 3c). At 80°C, again, phages CPLS29, CPLS32, and CPLS38 retained activity, but their plaquing efficiencies were now reduced by 50%, 75%, and 60%, respectively (Fig. 3d). No phages survived the 90°C treatment.

### Virulence assays with individual phages

**Fig 4:**
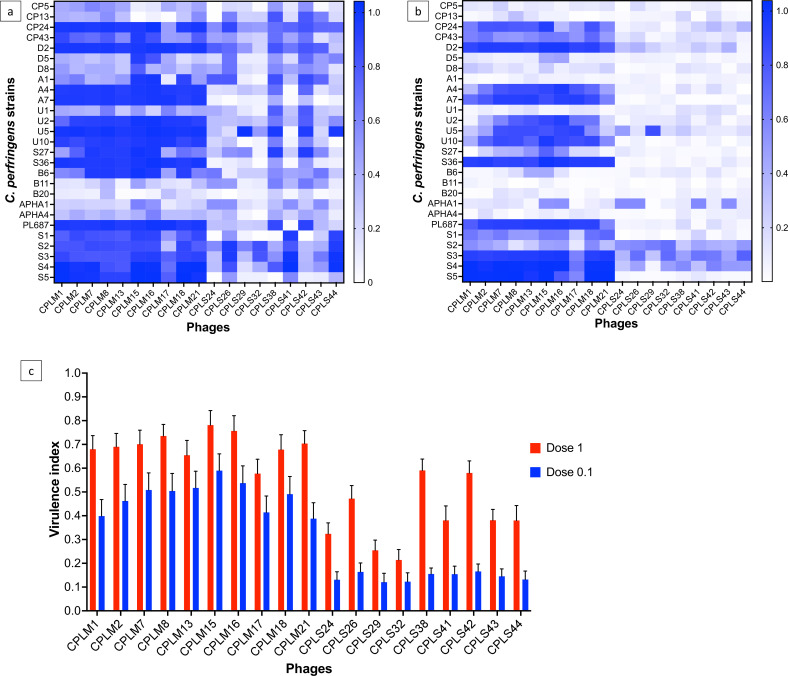
*V_i_* scores of 19 phages tested against 27 *C*. *perfringens* strains at doses (**a**) 1 and (**b**) 0.1. The bar to the right highlights the color designated to the *V_i_* scores, where 0 = no killing and 1 = maximum theoretical virulence. Graph (**c**) presents the average virulence index of individual phages on all tested strains, and error bars show the standard error of the mean.

Virulence assays were conducted with individual phages on 27 *C*. *perfringens* strains selected to include strains the phage(s) lyse, strains on which phage(s) produce hazy clearance, and strains phage(s) are unable to lyse. The host range of the 19 phages on the 27 *C*. *perfringens* strains selected is shown in Table S3. To screen for phage virulence, phages were tested at two doses of 1, which was equal to concentrations of *C. perfringens* at 3 × 10^7^ colony forming unit (CFU/mL) and phage at 3 × 10^7^ PFU/mL and dose 0.1, which was *C. perfringens* at 3 × 10^7^ CFU/mL and phage at 3 × 10^6^ PFU/mL concentration (10× less phages). Local virulence (*V_i_*) was used to score phage virulence (Fig. 4).

At dose 1, phages had higher *V_i_* scores across all tested strains than at dose 0.1, suggesting that they are more effective at lysing at a higher concentration (Fig. 4a and c). Similar to the host range analysis, CPLM phages had higher *V_i_* scores than CPLS phages. Overall, phage CPLM15 was the most virulent with *V_i_* scores above 0.9 against 18/27 *C. perfringens* strains (Table S4). Among the CPLS phages, CPLS38 and CPLS42 were the most virulent with *V_i_* scores above 0.5 against 19/27 and 17/27 strains, respectively. In comparison, phage CPLS32 was the least virulent and against 12/27 strains had *V_i_* scores between 0 and 0.1 (Table S4).

At a dose of 0.1, phages CPLM15 and CPLM16 had *V_i_* scores above 0.9 for 8/27 strains (Fig. 4b). For 17/27 *C*. *perfringens* strains, there was at least one phage that had *V_i_* scores of ~0.5. Phages CPLS24 and CPLS29 were the least virulent with *V_i_* scores between 0 and 0.1 against 20/27 strains.

### Five-strain *C. perfringens* infection models

To screen and compare the virulence of phage cocktails, we optimized a five-strain *C. perfringens* virulence model. We used the same model design used by the Animal and Plant Agency, UK, and commercial companies to experimentally induce NE in poultry in order to study the infection (16) as our objective was to develop laboratory models to test phage virulence that could subsequently inform on which phage cocktails are tested in poultry. The models we developed were a virulence assay and larva infection models. For both studies, we mixed strains S1, S2, S3, S4, and S5 at equal volumes to produce a mixed culture.

### Virulence assays with phage cocktails

**Fig 5:**
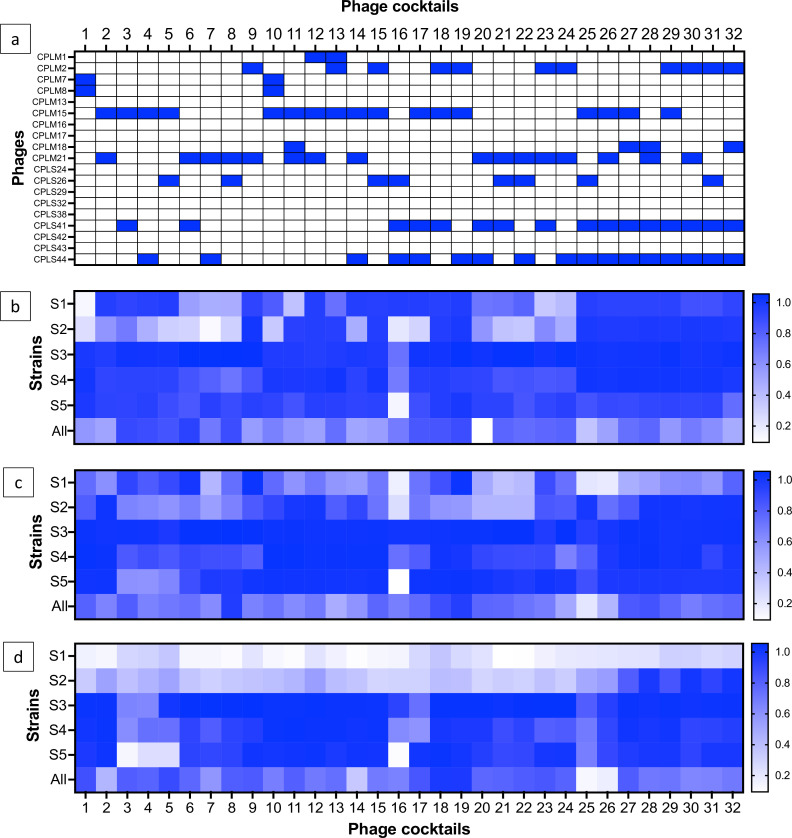
*V_i_* scores of 32 phage cocktails (**a**) on *C. perfringens* strains S1, S2, S3, S4, and S5 and mixed culture at doses (**b**) 1, (**c**) 0.1, and (**d**) 0.01. The bar to the right highlights the color designated to the *V_i_* scores, where 0 = no killing and 1 = maximum theoretical virulence.

Virulence assays were conducted to determine and compare the lytic activity of phage cocktails (16) (Fig. 5). Virulence was tested on individual strains S1, S2, S3, S4, and S5 and mixed culture to compare virulence on individual strains versus mixed cultures. Based on host range and virulence assays, individual phages were effective at lysing these five strains (Fig. 4; Tables S3 and S4). Thirty-two different phage cocktail combinations at doses of 1, 0.1, and 0.01 were tested to determine dose–response, and phage cocktails are listed in Table 2.

**TABLE 2:** List of phage cocktails tested in virulence assay studies

Phages included within the cocktail	Phage cocktail name
CPLM7 + CPLM8	C1
CPLM15 + CPLM21	C2
CPLM15 + CPLS41	C3
CPLM15 + CPLS44	C4
CPLM15 + CPLS26	C5
CPLM21 + CPLS41	C6
CPLM21 + CPLS44	C7
CPLM21 + CPLS26	C8
CPLM2 + CPLM21	C9
CPLM7 + CPLM8 + CPLM15	C10
CPLM15 + CPLM18 + CPLM21	C11
CPLM1 + CPLM15 + CPLM21	C12
CPLM1 + CPLM2 + CPLM15	C13
CPLM15 + CPLM21 + CPLS44	C14
CPLM2 + CPLM15 + CPLS26	C15
CPLS26 + CPLS41 + CPLS44	C16
CPLM15 + CPLS41 + CPLS44	C17
CPLM2 + CPLM15 + CPLS41	C18
CPLM2 + CPLM15 + CPLS44	C19
CPLM21 + CPLS41 + CPLS44	C20
CPLM21 + CPLS26 + CPLS41	C21
CPLM21 + CPLS26 + CPLS44	C22
CPLM2 + CPLM21 + CPLS41	C23
CPLM2 + CPLM21 + CPLS44	C24
CPLM15 + CPLS26 + CPLS41 + CPLS44	C25
CPLM15 + CPLM21 + CPLS41 + CPLS44	C26
CPLM15 + CPLM18 + CPLS41 + CPLS44	C27
CPLM18 + CPLM21 + CPLS41 + CPLS44	C28
CPLM2 + CPLM15 + CPLS41 + CPLS44	C29
CPLM2 + CPLM21 + CPLS41 + CPLS44	C30
CPLM2 + CPLS26 + CPLS41 + CPLS44	C31
CPLM2 + CPLM18 + CPLS41 + CPLS44	C32

**Fig 6:**
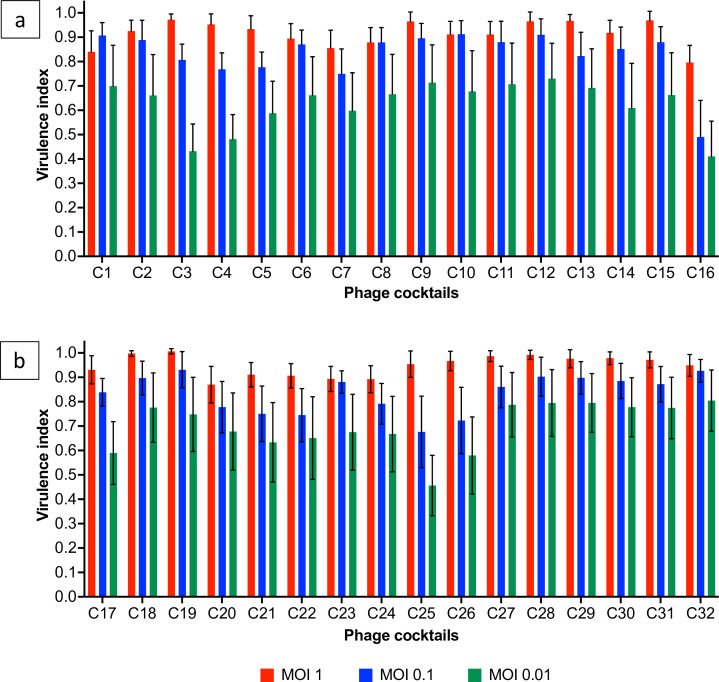
Average *V_i_* scores of C1–C16 (**a**) and C17–C32 (**b**) phage cocktails on strains S1, S2, S3, S4, and S5 and mixed culture at three doses. Error bars show the standard error of the mean.

Virulence assays highlighted that three-phage cocktails (C)18 and C19 and four-phage cocktails C27–C32 were consistently the most virulent combinations across all tested doses, all individual strains, and mixed cultures (Fig. 5 and 6). Both C18 and C19 included phages CPLM2 and CPLM15, but the third phage differed, with CPLS41 and CPLS44 being present in C18 and C19, respectively. These data suggest that a phage combination of CPLM2 and CPLM15 together in the cocktail is virulent. In comparison, the two-phage cocktails C3 and C4 were the least virulent based on *V_i_* scores. Both cocktails included CPLM15 with CPLM41 and CPLM44 as the second phage, respectively. This could suggest that the absence of CPLM2 was contributing to the reduction in virulence.

The virulent four-phage cocktails C27, C28, C29, C30, and C32 all included two CPLM and two CPLS, which could suggest that mixing these phages at equal volumes contributes to virulence. In comparison, there was a significant dose-dependent reduction in *V_i_* scores of the four-phage cocktail C25, which included CPLM15, CPLS26, CPLS41, and CPLS44 despite including the virulent CPLM15. This could suggest that the addition of CPLM15 to C25 did not improve virulence.

A dose–response reduction in *V_i_* scores was observed for all phage cocktails (Fig. 5 and 6). However, individual strains S3, S4, and S5 were the most susceptible to phage infection, and even at the lowest dose, 20/32 cocktails had *V_i_* scores above 0.8 against these strains versus 30/32 cocktails at a dose of 1. In comparison, strains S1 and S2 were less susceptible to phage infection, and at a dose of 0.01, 16/32 cocktails had *V_i_* scores below 0.2 on these strains. Despite this, overall *V_i_* scores were consistently high against mixed cultures, and there was a dose-dependent reduction in *V_i_* scores.

### Infection dynamics of phage cocktails in *G. mellonella*

Using the mixed culture, we developed a larva infection model in which the efficacy of 16 phage cocktails was tested. The cocktails tested included a mix of virulent and less virulent cocktails based on their *V_i_* scores to determine if their virulence was comparable in the larva model. Phage efficacy was also tested at the same three doses of 1, 0.1, and 0.01 as the virulence assays to determine dose–response (Table 3).

**TABLE 3:** List of larva groups used to assess phage efficacy for each cocktail tested

Larva group	Number of larvae included	Description
1	20	Injected with phage cocktail at dose 10^6^ PFU/larva (dose 1) and 1 hour later infected with *C. perfringens* mixed culture at 10^6^ CFU/larva
2	20	Injected with phage cocktail at dose 10^5^ PFU/larva (dose 0.1) and 1 hour later infected with *C. perfringens* mixed culture at 10^6^ CFU/larva
3	20	Injected with phage cocktail at dose 10^4^ PFU/larva (dose 0.01) and 1 hour later infected with *C. perfringens* mixed culture at 10^6^ CFU/larva
4	20	Injected with phage cocktail at dose 10^5^ PFU/larva and 1 hour later injected with PBS
5	20	Injected with phosphate-buffered saline (PBS) and 1 hour later infected with *C. perfringens* mixed culture at 10^6^ CFU/larva
6	20	Injected with PBS
7	20	Uninfected larvae (no phage and no *C. perfringens* mixed culture infection)

First, the model was optimized to determine the median lethal *C. perfringens* dose (lethal dose - LD_50_), which was 10^6^ CFU/larva for the mixed culture. This dose resulted in the survival of 50% of larvae after 24 hours, and after 72 hours, the survival rates dropped to 20%; thus, this dose was selected for subsequent infection studies (Fig. S1a). The LD_50_ was also tested for all five strains individually, and they had statistically comparable virulence to each other and the mixed culture (Fig. S1b through f).

**Fig 7:**
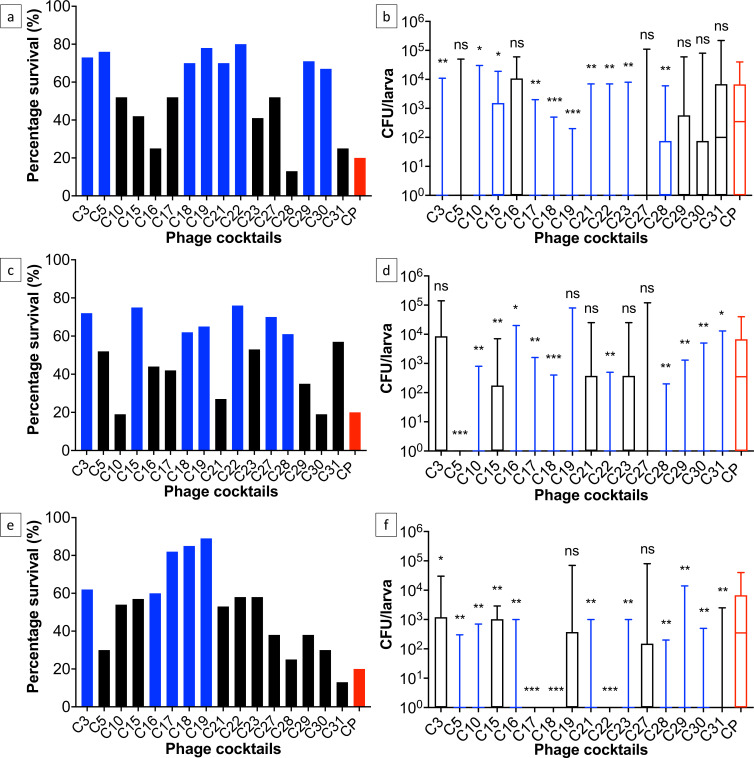
Efficacy of 16 phage cocktails in improving survival and reducing *C. perfringens* colonization in infected larvae at doses 1 (**a and b**), 0.1 (**c and d**), and 0.01 (**e and f**). Graphs (a, c, and e) show larva survival 72 hours after infection, and blue bars highlight over 60% survival, black bars highlight below 60% survival, and the red bar shows the survival of infected larvae without phage. Boxplots (b, d, and f) show *C. perfringens* abundance after 72 hours. Student *t*-tests were used to determine statistically significant differences in *C. perfringens* abundance between infected larvae without phage treatment (red bars) and treatment with phage cocktails. *P*-values are displayed on graphs (ns > 0.05, ^*^P < 0.05, ^**^P < 0.01, and ^***^P < 0.001).

Phage cocktails C10, C15, and C17 had a significant dose-dependent reduction in *V_i_* scores in the virulence assays and C18 and C22, which were the most virulent based on *V_i_* scores were the most effective at reducing *C. perfringens* colonization in larvae (*P* < 0.05) across all doses (Fig. 7). However, paired with larva survival data, only C18 (CPLM2, CPLM15 and CPLS41) improved larva survival above 60% across all doses after 72 hours whereas in the absence of phages, the survival rates of infected larvae were only 20%. Furthermore, with prophylactic administration of C18, in ~92% of infected larvae, *C. perfringens* was not re-isolated regardless of the dose. Average *C. perfringens* counts were 1.5 × 10^1^ CFU/larva across doses of 1 and 0.1 after 72 hours, but at the lowest dose, *C. perfringens* was not re-isolated from any infected larvae. In comparison, average *C. perfringens* counts in infected larvae in the absence of phage treatment was ~6.6 × 10^3^ CFU/larva and was re-isolated from 80% of infected larva (Fig. 7; Table 4).

**TABLE 4:** Number of larva that were positive for *C. perfringens* for each phage cocktail after 72 hours of infection

	Larva positive for *C. perfringens* per cocktail (n = 20)																
Dose	C3	C5	C10	C15	C16	C17	C18	C19	C21	C22	C23	C27	C28	C29	C30	C31	CP^*a*^
**1**	2	3	2	7	8	3	1	1	2	2	2	3	5	7	5	10	15
**0.1**	7	0	2	6	2	3	1	3	6	2	6	3	2	2	2	3	15
**0.01**	5	2	3	6	2	0	0	5	3	0	3	5	2	2	2	3	15

^*a*^ Larva infected with mixed *C. perfringens* culture only in the absence of phage.

C27 was the least effective cocktail in the larva infection study despite the virulence assays identifying the cocktail as having high *V_i_* scores against the mixed culture and individual strains. C27 did not significantly reduce *C. perfringens* colonization in infected larvae at all three doses, and average counts were 7 × 10^3^ CFU/larva after 72 hours. However, C27 did improve survival of infected larvae by 20%–55% at all doses.

Phage cocktails C5, C16, C28, C29, C30, and C31 were more efficacious at reducing *C. perfringens* colonization at lower doses of 0.1 and 0.01 than at 1 after 72 hours. At dose 1, average *C. perfringens* abundance was 5 × 10^3^ CFU/larva, but at lower doses, average abundance significantly reduced to 5 × 10^1^ CFU/larva, and *C. perfringens* was re-isolated from only 30% of infected larvae (Fig. 7; Table 4).

## DISCUSSION

In this study, we isolated 32 phages and showed that they variably infect globally prevalent *C. perfringens* strains. We found CPLM phages, which have myovirus morphology, to be the broadest acting in lysing over 80% of *C. perfringens* strains that are representative of circulating global strains on farms. In particular, phages CPLM7, CPLM8, CPLM15, CPLM16, and CPLM17 had the broadest host ranges and lysed over 90% of strains and, therefore, may each have therapeutic potential. In comparison, the CPLS siphovirus and CPLP podovirus morphologies had narrower host ranges, which suggests there potentially could be a link between phage morphology and infectivity. Previous work has noted that *C. perfringens* podoviruses, for example, phages DCp1 and CPQ1, have narrow host ranges and infected 9.3% (5/54) and 24% (6/25) strains tested, respectively (17, 18), in accordance with the work presented here.

Host range screening identified 19/32 phages that could infect over 80% of *C. perfringens* strains, making them ideal candidates for therapeutic use. These phages were characterized further and found to be stable from pHs 4–11. pH stability is critical as if phages are administered orally, they will be exposed to pHs ranging from 1 to 7 in the digestive tract (19). Low pH in particular could inactivate phages. However, a recent poultry study using phages SPFM10 and SPFM14 (which had similar pH stability as the current phages) resulted in their recovery from fecal samples suggesting that the digestive tract pH changes did not impact their activity (20). In addition, we determined temperature stability and noted that these phages are stable up to 60°C, which is similar to other characterized *C. perfringens* phages (17, 21, 22). However, phages CPLS29, CPLS32, and CPLS38 were more thermostable and retained activity after being incubated for 1 hour at 70°C and 80°C. Increased heat stability increases the options of how phages are manufactured (e.g., by spray drying) and ultimately delivered (via incorporation into steam-pelleted feed) to livestock. Drying phages can also extend their shelf life and make them easier to transport (18).

Virulence assays highlighted that phage CPLM15 was the most virulent and also had the broadest host range. In comparison, phages CPLS24 and CPLS29 had the lowest *V_i_* scores across the strain collection despite being able to infect 80% of strains in the host range screening. Our data set suggests that host range screening does not consistently correlate with virulence data, and both methods should be used to measure phage virulence. A similar finding was also observed during screening and virulence testing of *Escherichia coli* and *Klebsiella* phages (23).

We found that overall virulence was increased when phages were combined as cocktails. Phage cocktails C18 and C19 had high *V_i_* scores across the tested doses, which highlights that lowering the dose did not impact their virulence. Both cocktails included phages CPLM2 and CPLM15, which individually had high *V_i_* scores against *C. perfringens* phages. The addition of a third phage CPLS41 and CPLS44 to C18 and C19, respectively, improved their virulence. It is likely that there is synergy between the phages in the cocktails, which contributed to their virulence and their efficacy even at lower doses. The synergy between the phages could be related to one phage improving adsorption, the rate of infection, progeny production, or the connecting time between infection and release of the progeny of the other phage, which was experimentally shown to be the case between two virulent *E. coli* phages (24).

In comparison, cocktails C4 and C25 showed high virulence at dose 1, but at lower doses, their *V_i_* scores declined. C4 included phages CPLM15 and CPLS44, which were two of the phages present in the virulent cocktail C19. This suggests that in the absence of CPLM2, there may be competitive inhibition and a lack of synergy between phages CPLM15 and CPLS44 alone. C25 included phages CPLM15, CPLS26, CPLS41, and CPLS44, which were also present in C18 and C19, but the cocktail had low *V_i_* scores. This again could be due to a lack of synergy or inhibition. Antagonistic interactions could be due to host infection competition between phages, whereby infection of the same host cell by multiple phages leads to competition for cellular resources, causing a reduction in the fitness of individual phages (25). Thus, our findings suggest the importance of screening multiple phage cocktail combinations as phage synergy and antagonistic interactions may play a part in its efficacy.

To further probe phage virulence, we developed a multi-strain larva infection model. Phage cocktails were administered prophylactically as studies suggest this allows their distribution throughout the hemolymph before the introduction of the pathogen, which could limit their ultimate colonization (26, 27). Their impact on larva survival and reduction in *C. perfringens* colonization varied between phage cocktails. Consistent with the virulence assays, C18 (CPLM2, CPLM15, and CPLS41) significantly improved larva survival and reduced *C. perfringens* abundance across all three doses within the multi-strain model. Thus, C18 could be a viable phage cocktail to test in challenge poultry studies. In the virulence testing, C19 was also identified as an optimal cocktail; however, in the larva infection study, C19 did not consistently reduce *C. perfringens* colonization across all three doses but did improve the survival of infected larvae. Therefore, not all phage cocktails identified as virulent via *V_i_* screening are efficacious in more complex infection studies. Thus, our data set suggests that testing phage efficacy in a larva infection model may be beneficial as an additional virulence measure for phage cocktail comparisons.

In our data set, we identified 6/16 phage cocktails that were more efficacious at lower dosages, which could suggest that synergy between the phages in the cocktails improves when the phages are further diluted. The reason why the lower dose was more effective is unclear. One possible explanation could be that with the lower phage dose, there is a slower rate of bacterial lysis, which could allow for increased localized phage infection and replication before their clearance by the immune system.

In conclusion, in this study, we have isolated a collection of *C. perfringens* phages and tested their efficacy using multiple methods to design a virulent phage cocktail. Our data set highlights that phage efficacy should be tested using various methods that include host range analysis against global target strains and local virulence and determine their efficacy in the larva challenge studies that mimic infection in poultry. The models each have their own strengths and weaknesses but collectively pre-screen optimal phage or phage cocktail to inform downstream animal studies.

## MATERIALS AND METHODS

### Bacterial strains and growth conditions

Ninety-seven *C*. *perfringen*s strains were used, all of which were originally isolated from infected chickens and include toxin type A and G strains (Table S1). Thirty-six *C*. *perfringen*s strains were from Belgium, eleven strains from Denmark, seven from Australia, and ten from USA, which were all isolated by Dr Filip Van Immerseel research group at Ghent University, Belgium. Four strains were isolated by Dr Chung-His Chou at the National Taiwan University, Taiwan; 16 strains were from Brazil and 13 strains from the Animal and Plant Health Agency, UK.

All *C. perfringens* strains were stored in 1 mL tubes containing 50% glycerol (Abtex Biologicals Ltd., UK) at −80°C. *C. perfringens* strains were grown on selective medium Perfringens TSC agar base (Oxoid, UK) overnight at 37°C under anaerobic conditions (10% H_2_, 5% CO_2_, and 85% N_2_, Don Whitley Scientific, West Yorkshire, UK), on which they produce black colonies. For liquid cultures, single colonies were subcultured in brain heart infusion (BHI) broth overnight at 37°C under anaerobic conditions. BHI broth was stored under anaerobic conditions at 37°C, 24 hours before use, to pre-reduce the media to anaerobic conditions.

### Phage isolation, purification, and propagation

One hundred fifty-nine cow, chicken, and sheep fecal samples and silage, slurry, and waste-water samples were collected across the UK. Phages isolated from these samples are listed in Table 1. All samples were processed using the same enrichment protocol: 1 g of material was mixed with pre-reduced medium of 9 mL of BHI broth and 1 mL of salts (0.01 M CaCl_2_, 0.4 M MgCl). 100 µL of overnight *C. perfringens* cultures was added to the broth, and to maximize phage isolation, the same environmental sample was aliquoted and enriched with 26 different *C. perfringens* host strains individually. Bacterial strains added to the medium without the addition of environmental samples were included as controls, and all control samples were negative for phage. Samples were then incubated anaerobically at 37°C for 48 hours, after which they were centrifuged at 4,200 × *g* for 15 minutes, filtered using a 0.22-µm pore size syringe filter, and stored at 4°C until use.

The small drop plaque assay method was used to screen for phages in the filtrates (28). Briefly, 100 µL of the filtrate was mixed with 300 µL of an overnight *C. perfringens* culture and 3 mL of top agar and BHI 0.5% (wt/vol) agar supplemented with 10% of salts. The mixture was then poured onto BHI 1% (wt/vol) agar plates, and plates were stored overnight under anaerobic conditions at 37°C. Each filtrate was screened for phage against the *C. perfringens* strain library. After overnight incubation, plates were checked for phage lysis, which was viewed as a clear lawn or individual plaques. If the former was found, the filtrate was serially diluted 10-fold and re-plated to isolate individual plaques. Plaques were picked using 300-µL pipette tips, stored in 0.5-mL BHI broth, and centrifuged at 21,000 × *g* for 5 minutes. The supernatant was then used for the next round of phage amplification and purification. The process was repeated five times to produce clonal phage stocks (29).

To increase phage volumes, phages were propagated in a liquid medium. To 9 mL of pre-reduced BHI broth and 1 mL of salts, 100 µL of an overnight *C. perfringens* culture was added and grown to optical density (OD_600_) 0.2. After which, 250 µL of phage was added. The mixture was incubated overnight under anaerobic conditions at 37°C, centrifuged at 4,200 × *g* for 10 minutes, filtered using 0.22-µm pore size filters, and stored at 4°C till use. Phage lysate was serially diluted 10-fold to determine titer as previously described (29).

### Transmission electron microscopy

TEM imaging of the *C. perfringens* phage collection was conducted at the University of Leicester, UK, by Natalie Allcock as previously described (20). Briefly, phages were negatively stained with 1% (wt/vol) uranyl acetate on 3-mm carbon-coated copper grids, and the JEM-1400 TEM (Jeol UK Ltd., UK), which had an accelerating voltage of 120 kV, was used to visualize phages. The Megaview III digital camera (EMSIS, Germany) was used to collect digital images.

### Phage host range

*C. perfringens* strains were grown overnight on Perfringens TSC agar, after which single colonies were inoculated in BHI broth and grown anaerobically at 37°C for 3 hours. As described above, 300 µL of culture was mixed with 3 mL of top agar and poured onto BHI 1% (wt/vol) agar circular plates. The lawn was left to set for 5 minutes; then, 10 µL of phage at titer 5 × 10^8^ PFU/mL was spotted on the bacterial lawn in triplicate. Spots were dried, and plates were incubated anaerobically at 37°C overnight. The appearance of phage spots was scored based on three criteria: complete lysis (clear spot), hazy lysis (not completely clear spot), and no phage clearance (Table S1).

### pH and temperature stability

For pH stability tests, 50 µL of phage lysate was mixed with 450 µL of SM buffer (100 mM NaCl, 8 mM MgSO_4_·7H_2_O, and 50 mM Tris-Cl) at pHs 1, 2, 3, 4, 5, 6, 7, 8, 9, 10, 11, 12, 13, and 14. The phage with buffer samples was incubated for 1 hour at room temperature. For the temperature stability assays, phage lysates were incubated at temperatures 4°C, 40°C, 50°C, 60°C, 70°C, 80°C, and 90°C for 1 hour.

After incubation at different pHs or temperatures, phages were serially diluted with 10-fold dilutions and were spotted in triplicate on lawns with their host bacteria. Plates were incubated overnight anaerobically at 37°C. Three biological replicates, each with three technical replicates, were performed. The phage titers after incubation at different pHs were compared to the original phage titer and expressed as plaquing efficiency, where 0 = phage titer has dropped to below detection limit and 1 = the pH/temperature did not alter the phage titer. The phage titers after incubation at different temperatures were compared to phage titers at 4°C and expressed as plaquing efficiencies.

### Virulence assays

Virulence assays were conducted using the BMG Labtech SPECTROstar Omega plate reader (Germany) with a flat bottom 96-well plate (Sarstedt, Germany). To set up 96-well plates, 100 µL of an overnight *C. perfringens* culture grown anaerobically at 37°C was added to pre-reduced 10 mL of BHI broth and grown anaerobically to OD 0.2 (~3 × 10^7^ CFU/mL). To test phage virulence on the mixed strain culture, strains S1, S2, S3, S4, and S5 were grown individually to OD 0.2 in liquid broth. After which, they were mixed at equal volumes to make the mixed strain culture with a final bacterial concentration of ~3 × 10^7^ CFU/mL. To *C. perfringens* cultures at OD 0.2, phage(s) were added at volumes dependent on the concentration and dose being tested. 200 µL of the phage culture mix was added to the 96-well plates, and three technical replicates were included. The 96-well plate was sealed with gas-permeable parafilm M (Amcor, UK) and placed within the plate reader. OD_600_ readings were taken every 5 minutes for 6 hours for individual phages and 12 hours with phage cocktails. Three biological replicates were conducted for each bacteria and phage combination tested. For each run, controls in triplicate were also included which were bacterial culture alone, BHI broth with phage(s), and BHI broth alone.

A quantitative method to measure phage virulence was used to analyze the output OD data (14). The local virulence (*V_i_*) score was calculated using the equation: *V_i_* = 1 – *A_i_*/*A_o_. A_i_* is the area under the curve of phage-infected bacterial cultures, and *A_o_* is phage-free bacterial cultures. Local virulence scores were between 0 and 1, where 0 = no killing and 1 = maximum theoretical virulence.

### Testing efficacy of phage cocktail in *Galleria mellonella* infection studies

*G. mellonella* larvae were purchased from Live Food UK Ltd. (Rooks Bridge, UK). For all experiments, larvae weighing between 0.25 and 0.3 g were selected and surface-sterilized with 70% ethanol before infection studies.

To determine the LD_50_ to kill half the number of infected larvae, five toxin A type *C. perfringens* strains (S1, S2, S3, S4, and S5) were mixed at equal volumes and diluted to doses 10^7^, 10^6^, 10^5^, 10^4^, and 10^3^ CFU/larva (21). The strains were prepared individually by growing them in 10 mL BHI cultures overnight, after which they were centrifuged at 4,200 × *g* for 10 minutes, the supernatant was decanted, and the bacterial pellets were re-suspended in 10 mL PBS. This step was repeated twice after which the strains were mixed at equal volumes and diluted to the required dose for the mixed culture. For each *C. perfringens* dose tested, 20 larvae were infected at a volume of 10 µL in the larva’s right posterior proleg via the Hamilton syringe pump. Larvae were incubated at 37°C for 72 hours, and survival was monitored every 24 hours. Larva survival was monitored by examining their responsiveness to touch, and if they were unresponsive, they were recorded as dead. Also, larvae that changed color from light brown to black were marked as dead.

For larva infection studies, previously published protocols were followed with some modifications (30–32). Phage cocktails were administered 1 hour before infection with *C. perfringens* at a volume of 10 µL, which was injected in the larva’s left posterior proleg via the Hamilton syringe pump. After phage and *C. perfringens* infection, larvae were incubated at 37°C for 3 days, and larva survival was recorded every 24 hours. On the last day of the study, all larvae were dissected to extract their hemolymph to determine *C. perfringens* counts. The extracted hemolymph was mixed with 1 mL PBS and vortexed for 30 seconds. For *C. perfringens* counts, the mix was diluted 10-fold and spot-tested on selective *Perfringens* TSC agar base plates, and colonies were counted to determine the CFU counts. With this method, the minimum detection limit was 100 CFU/larva, and if no colonies were detected on the lowest dilution (undiluted sample), CFU/larva counts were recorded as 0. Three technical repeats were conducted for *C. perfringens* counts, and three biological repeats were conducted for each phage cocktail combination tested.

For each phage cocktail combination tested in the infection model, 20 larvae were used. Control groups were included in all experiments, which are listed in Table 3, and again, for each group, 20 larvae were included.

### Statistical analysis

To analyze the pH and temperature stability data, Prism version 9.0.2 (La Jolla, CA, USA) was used. Data plotted on bar graphs show averages with ±standard error of the mean from three biological replicates. The same software was used to calculate the area under the curve to determine the local virulence.

For all larva studies, three biological studies were conducted, and the collective data were used to plot larva survival using Prism version 9.0.2 with the Kaplan–Meier method. The Log rank Mantel–Cox test survival rates were used to assess differences in survival rates. To determine if *C. perfringens* abundance was statistically significant between phage cocktails tested, Student *t*-tests were conducted. Values of *P* < 0.05 were considered as statistically significant.

## SUPPLEMENTAL MATERIAL

Figure S1 spectrum.03784-23-s0001.docx LD_50_.

Supplemental tables spectrum.03784-23-s0002.xlsx Tables S1 to S5.

ASM does not own the copyrights to Supplemental Material that may be linked to, or accessed through, an article. The authors have granted ASM a non-exclusive, world-wide license to publish the Supplemental Material files. Please contact the corresponding author directly for reuse.
