# Supplementary material for: Broad host range phages target global Clostridium perfringens bacterial strains and clear infection in five-strain model systems
Source: Microbiol Spectr. 2024 Mar 21;12(5):e03784-23. doi: 10.1128/spectrum.03784-23 (PMC11064546; doi:10.1128/spectrum.03784-23)
Supplement: Figure S1 — LD50. [file spectrum.03784-23-s0001.docx]

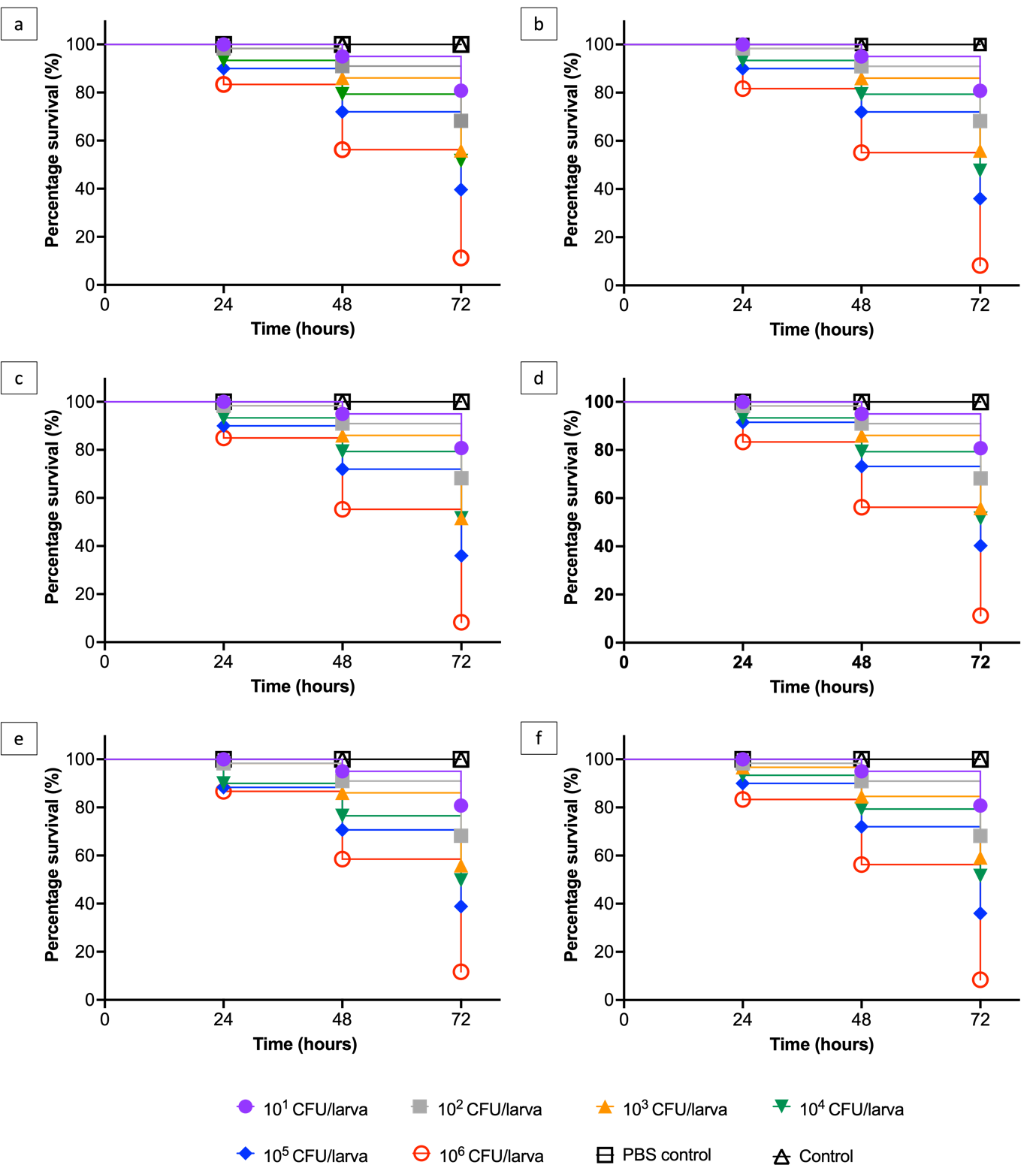


**Supplementary Figure 1**. Larvae were infected with different doses of (a) mixed culture, (b) S1, (c) S2, (d) S3, (e) S4 and (f) S5 to determine LD_50_. The graph shows larvae survival at 24, 48 and 72 hours after infection.
